# Efficient *in vitro* generation of functional thymic epithelial progenitors from human embryonic stem cells

**DOI:** 10.1038/srep09882

**Published:** 2015-06-05

**Authors:** Min Su, Rong Hu, Jingjun Jin, Yuan Yan, Yinhong Song, Ryan Sullivan, Laijun Lai

**Affiliations:** 1 Department of Allied Health Sciences, University of Connecticut, Storrs, CT; 2 Guiyang Medical College, Guizhou, China; 3 Fujian Academy of Medical Sciences, China; 4 University of Connecticut Stem Cell Institute, University of Connecticut, Storrs, CT

## Abstract

Thymic epithelial cells (TECs) are the major components of the thymic microenvironment for T cell development. TECs are derived from thymic epithelial progenitors (TEPs). It has been reported that human ESCs (hESCs) can be directed to differentiate into TEPs *in vitro*. However, the efficiency for the differentiation is low. Furthermore, transplantation of hESC-TEPs in mice only resulted in a very low level of human T cell development from co-transplanted human hematopoietic precursors. We show here that we have developed a novel protocol to efficiently induce the differentiation of hESCs into TEPs *in vitro*. When transplanted into mice, hESC-TEPs develop into TECs and form a thymic architecture. Most importantly, the hESC-TECs support the long-term development of functional mouse T cells or a higher level of human T cell development from co-transplanted human hematopoietic precursors. The hESC-TEPs may provide a new approach to prevent or treat patients with T cell immunodeficiency.

T cells play a critical role in the immune system, providing protection against infections and cancers. The thymus is the primary organ for T cell generation. T cell development in the thymus depends on the thymic microenvironment, of which TECs are the major component^1^,^2^,^3^. TECs can be divided into cortical TECs (cTECs) and medullary TECs (mTECs); the former mediate positive selection; and the latter are involved in the negative selection by which potentially autoreactive T cells are deleted. The relevance of TECs to T cell development is demonstrated by the fact that abnormal TEC development results in autoimmunity and immunodeficiency^1^,^2^,^3^. Furthermore, a decrease in TEC number results in a reduced number of T cells^4^.

Studies have shown that TECs undergo degeneration over time, which is believed to be one of the major factors responsible for age-associated thymic involution^2^,^5^,^6^,^7^. Moreover, TECs can be injured by radiation, chemotherapy, infection, and graft-versus-host disease following bone marrow (BM) transplantation^8^,^9^,^10^. Therefore, approaches to restore or regenerate TECs should also result in enhanced generation of T cells.

We have reported that mESCs can be selectively induced to differentiate into TEPs *in vitro*^11^,^12^. When transplanted into mice, the mESC-TEPs further develop into cTECs and mTECs, reconstitute normal thymic architecture, and promote T cell regeneration, leading to an increased number of functional T cells in peripheral lymphoid organs^11^,^12^.

Recently, it has been reported that hESCs can also be induced to differentiate into TEP-like cells *in vitro*^13^,^14^,^15^. However, generation of hESC-TEPs by these protocols lacks efficiency. Furthermore, although hESC-TEPs in nude mice further developed into TECs that supported mouse T cell development, a progressive decline in the number of T cells was observed, and T cell regeneration was not sustained over 22 weeks^13^. In addition, only one report showed that hESC-TECs could support human T cell development from co-transplanted human hematopoietic precursors, but the percentage of newly generated human T cells in the peripheral blood of the recipient mice was very low^14^.

Here we report a new protocol for directing the differentiation of hESCs into TEPs *in vitro*. The hESC-TEPs, when transplanted into athymic nude mice, develop into both types of TECs that support a long-term generation of functional T cells. Most importantly, the hESC-TECs can also support a higher level of functional human T cell development from co-transplanted human hematopoietic precursors.

## Results

### Inducing the differentiation of hESCs into TEPs *in vitro*

**Figure 1:**
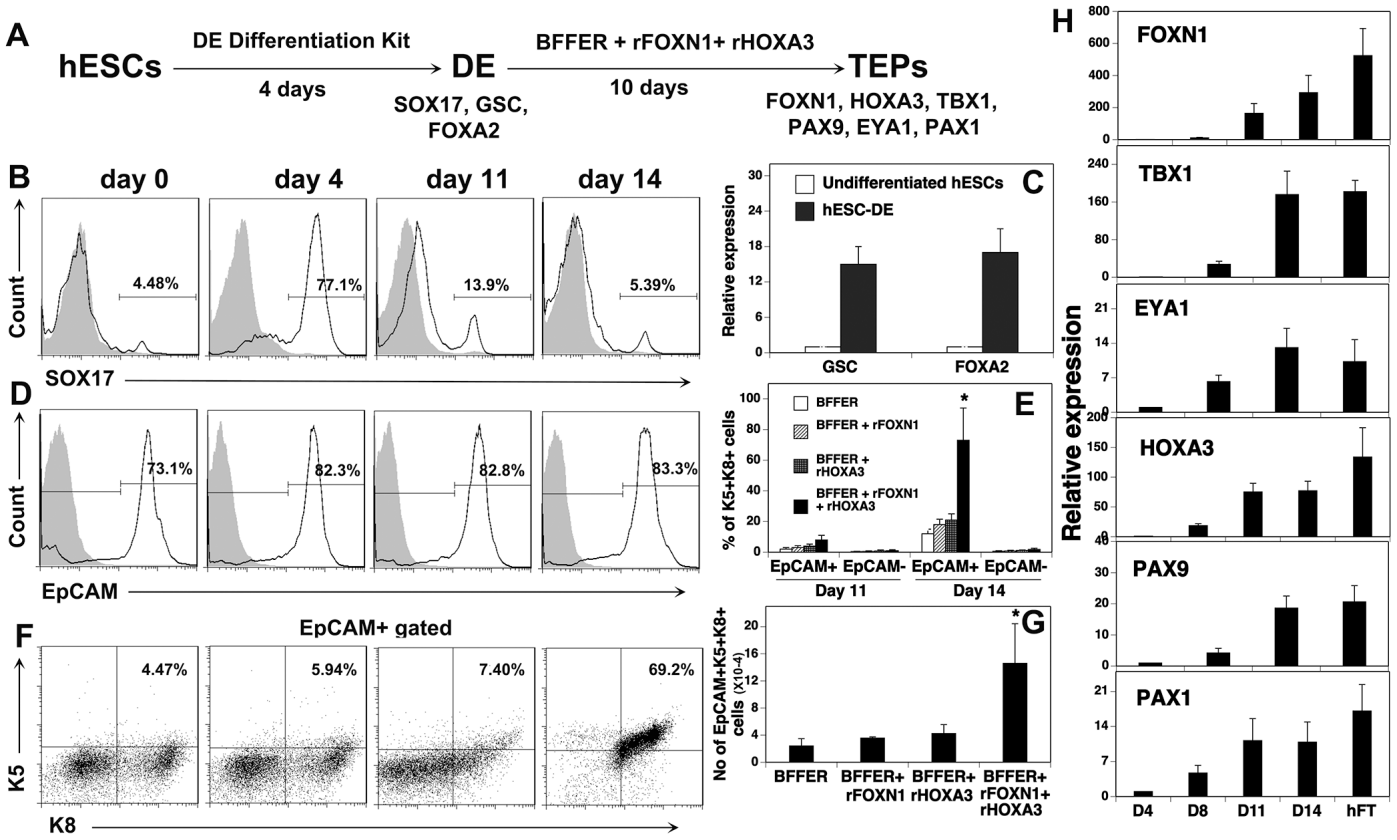
Generation of TEPs from hESCs *in vitro*. (A) Schematic of the differentiation protocol. hESCs were induced to generate DE that further developed into TEPs in the presence of BMP4 + FGF7 + FGF10 + EGF + RA (BFFER), rFOXN1 (100 ng/ml) and rHOXA3 (200 ng/ml). The protein and/or genes that were examined for the expression are also shown. (B) Flow cytometric analysis of the expression of SOX17 at day 0–14 hESC-derived cells. The gray filled lines are isotype controls. (C) qRT-PCR analysis of the expression of *GSC* and *FOXA2* by hESC-DE. Data are presented as relative levels of expression in hESC-derived cells versus undifferentiated hESCs. (D, E) Representative flow cytometric profiles of the expression of (D) EpCAM, and (E) K5 and K8 by EpCAM^+^ cells by days 0–14 hESC-derived cells that had been cultured with BFFER, rFOXN1 and rHOXA3. (F) Percentage of cells that co-expressed K5 and K8 at day 11 and 14 hESC-derived EpCAM^+^ and EpCAM^−^ cells. (G) The number of EpCAM^+^K5^+^K8^+^ putative TEPs in day 14 hESC-derived cells that had been cultured with BFFER, rFOXN1 and/or rHOXA3. * p < 0.05 compared with the culture containing BFFER only. (H) Kinetics analysis of the expression of *FOXN1, HOXA3, TBX1, PAX9, EYA1*, and *PAX1* in the hESC-derived cells from cultures containing BFFER + rFOXN1 + rHOXA3 by qRT-PCR. Data are presented as relative levels of expression on days 8, 11 and 14 hESC-derived cells versus day 4 hESC-DE. hFT was used as a positive control. The data are Mean ± SD from 3 independent experiments.

Figure 1A is a schematic of our differentiation protocol. Because the epithelial compartment of the thymus originates from the endoderm^1^,^2^,^16^, we first induced the differentiation of hESCs into definitive endoderm (DE) by the Human Pluripotent cell-derived Endoderm Differentiation Kit (R&D Systems) that contains specially formulated media supplements and growth factors including Activin A and Wnt-3a. Four days later, the cells were analyzed for the expression of the DE markers SOX17, goosecoid (GSC), and FOXA2. Flow cytometric analysis showed that more than 72% of the hESC-derived cells expressed SOX17 (Figure 1B). Real-time quantitative RT-PCR (qRT-PCR) analysis showed that the expression of the *GSC* and *FOXA2* genes was significantly increased as compared to undifferentiated hESCs (Figure 1C). These results indicate that the DE has been generated from hESCs.

We then directed the differentiation of the hESC-DE into TEPs. We have previously reported that the combination of fibroblast grown factor (FGF) 7, FGF10, Epithelial growth factor (EGF), and bone morphogenetic protein 4 (BMP-4) induces the differentiation of mouse ESCs into TEPs^11^,^12^. We added these growth factors to the hESC cultures. Because it has been reported that retinoic acid (RA) can enhance the development of TEPs from hESCs^13^,^14^, we also added RA. It is well known that FOXN1 is a pivotal regulator of thymic epithelium development and identity^15^,^17^,^18^,^19^. We have cloned and expressed recombinant (r) FOXN1 protein fused with the HIV transactivator of transcription (TAT) protein transduction domain (PTD) (amino acids 47–57) (Song Y et al. submitted for publication). It has been reported that TAT PTD mediated protein transduction with high efficiency in hESCs^20^. We have shown that rFOXN1 protein, when added into culture medium, can translocate from the cell surface into the cytoplasm and nucleus (Song Y et al. submitted for publication). Some of the cultures also received rFOXN1 (50–500 ng/ml). HOXA3 has been proposed to be the earliest regulator for thymus organogenesis^17^. It has been shown that the third α helix of the homeodomain can direct internalization of HOXA3 protein via receptor-independent passive translocation into cells^21^,^22^. We cloned and expressed the HOXA3 gene to produce a rHOXA3 protein that was confirmed by Western blot (see Supplemental Fig. S1 online). Some of the cultures additionally received rHOXA3 (100–500 ng/ml).

We analyzed for the expression of EpCAM because it has been shown to be expressed by TEPs^23^. We found that 69–88% of day 0–14 hESC-derived cells that had been cultured with or without rFOXN1 and/or rHOXA3 expressed EpCAM, and the percentages of EpCAM^+^ cells did not significantly differ among groups (Figure 1D and data not shown). Studies have shown that K5 and K8 double positive (K5^+^K8^+^) cells contain or represent TEPs^24^,^25^,^26^,^27^,^28^. We then examined the expression of K5 and K8 from the hESC-derived cells. As shown in Figure 1E and F, the addition of rFOXN1 and/or rHOXA3 slightly increased the percentage of K5^+^K8^+^ cells in day 11 hESC-EpCAM^+^ cells, as did the addition of rFOXN1or rHOXA3 in day 14 hESC-EpCAM^+^ cells. However, the differences did not achieve statistical significance. In contrast, the addition of rFOXN1 and rHOXA3 resulted in a significant 5.8–6.5-fold increase in the percentage and number of K5^+^K8^+^ cells in day 14 hESC-EpCAM^+^ cells (Figure 1E–G), as compared to cultures without rFOXN1 and rHOXA3. The results indicate that the combination of rFOXN1 and rHOXA3 can enhance the generation of hESC-TEPs. Because the greatest number of EpCAM^+^K5^+^K8^+^ cells were generated when rFOXN1 and rHOXA3 were added at the concentrations of 100 ng/ml and 200 ng/ml, respectively (data not shown), we used these doses in the follow-up studies. In all of the culture conditions, few hESC-EpCAM^−^ cells were K5^+^K8^+^ cells (data not shown), indicating that hESC-TEPs were located in the EpCAM^+^ cells.

We also analyzed for the expression of the third pharyngeal pouch endoderm (PPE) and TEP related genes *FOXN1, HOXA3, TBX1, PAX9, EYA1*, and *PAX1* by qRT-PCR. A significantly enhanced expression of these genes in the hESC-derived cells was observed on day 11, and the expression levels of these genes in day 11 hESC-derived cells were comparable to those in day 14 hESC-derived cells (Figure 1H). The expression of these molecules was confirmed at the protein level (see Supplemental Fig. S2 online, and data not shown). This is in contrast to the observation that the most EpCAM^+^K5^+^K8^+^ cells were generated only after day 14 (Figure 1F, G). These results suggest that the third PPE was probably generated in our culture conditions on days 9–11, and the third PPE further developed into TEPs on day 14.

### hESC-derived TEPs develop into TECs *in vivo*

**Figure 2:**
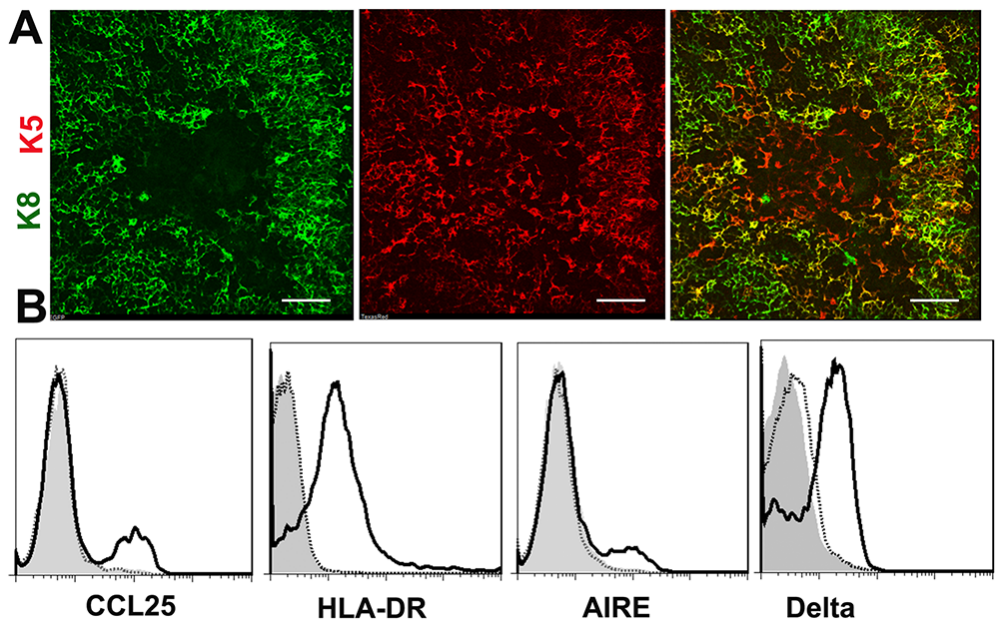
hESC-TEPs develop into TECs and form a thymic architecture *in vivo*. EpCAM^+^ and EpCAM^−^ cells were purified from day 14 hESC cultures, cell reaggregated *in vitro* and then transplanted under the kidney capsule of nude mice that had been irradiated (3 Gy) and injected i.p. with 25 μl anti-asialo-GM1. Twelve weeks later, the grafts were harvested. (A) Sectioned EpCAM^+^ grafts were immunofluorescently stained using K8 and K5 antibodies. A representative graft is shown. Scale bar = 100 μm. (B) Flow cytometric analysis of the expression of CCL25, HLA-DR, AIRE, and delta molecules by CD45^−^ cells in the EpCAM^+^ (solid lines) and EpCAM^−^ (dotted lines) grafts. Isotype antibodies were used as controls (gray filled lines).

To determine whether hESC-derived TEPs can develop into TECs *in vivo*, we purified hESC-derived EpCAM^+^ and EpCAM^−^ cells from the cultures. The cells were reaggregated *in vitro* and then transplanted under the kidney capsule of nude mice that had been irradiated and injected with anti-asialo-GM1 antibody to delete NK cells before the transplantation^29^. Three months later, the grafts were harvested and analyzed for structure by immunofluorescence. As shown in Figure 2A, discrete K8^+^K5^−^ cortical (green color) and K8^−^K5^+^ medullary (red color) areas were present in the EpCAM^+^ cell graft, suggesting that hESC-TEPs could generate cTECs and mTECs and form a thymic architecture *in vivo*. Some of the cells were K8^+^K5^+^ (yellow color), suggesting they were residual or self-replicating TEPs. In contrast, hESC-EpCAM^−^ cells could not form a thymic architecture *in vivo* (data not shown).We then analyzed the hESC-TECs for the expression of molecules critical for recruiting T cell precursors to and/or supporting T cell development in the thymus. As shown in Figure 2B, CD45^−^ cells that contain hESC-TECs in the EpCAM^+^ grafts expressed CCL25, HLA-DR, and AIRE molecules, whereas CD45^−^ cells from the EpCAM^−^ grafts did not express these molecules. We also examined the expression of delta-like Notch ligands with an antibody that recognizes epitopes common to the human delta-like 1–4^30^. We found that hESC-derived TECs also expressed these ligands. Collectively, these data indicate that hESC-TEPs *in vivo* can further develop into TECs that express molecules critical for T cell development.

### Transplantation of hESC-TEPs in nude mice results in long-term generation of mouse T cells

**Figure 3:**
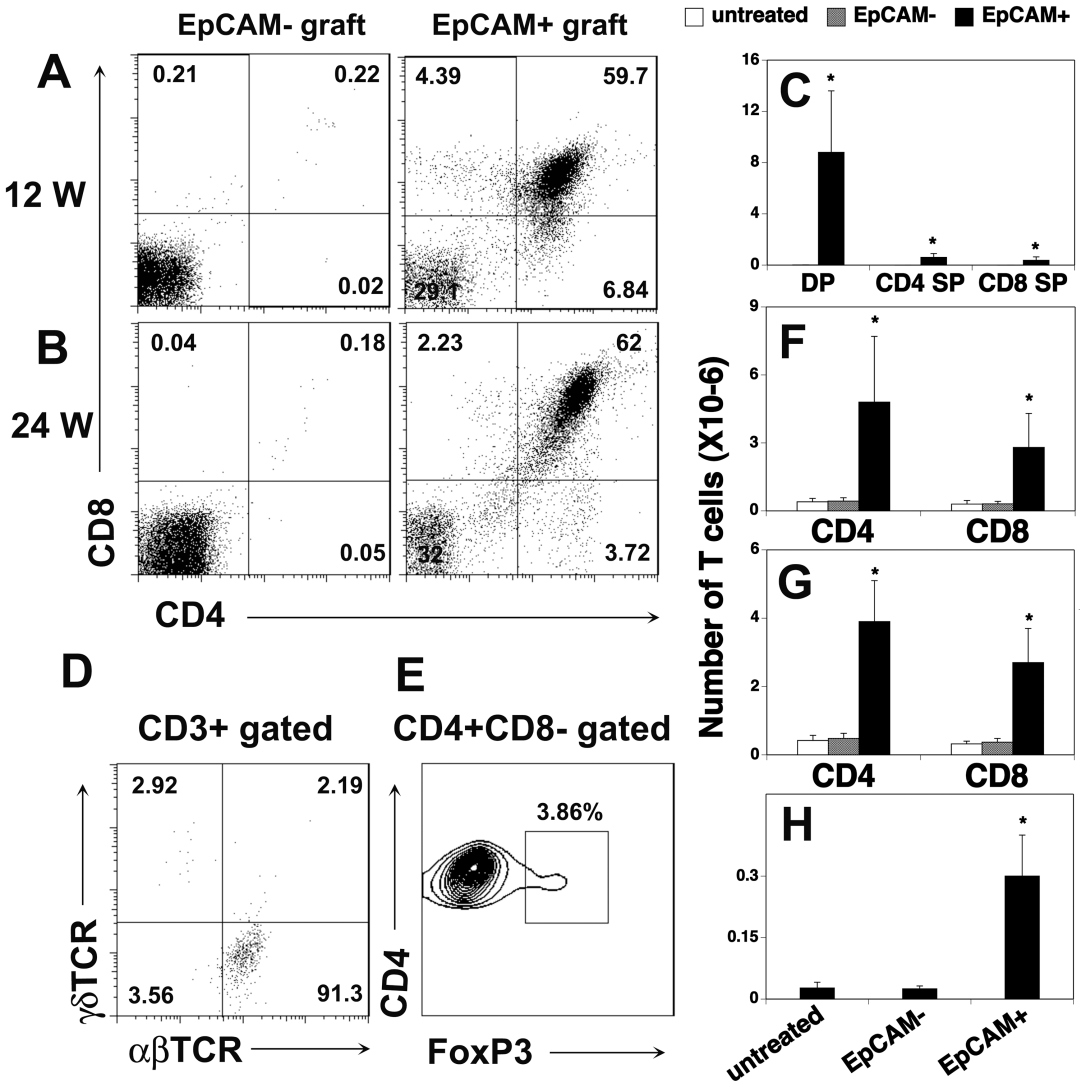
hESC-TECs support mouse T cell development *in vivo*. EpCAM^+^ and EpCAM^−^ cells were purified from day 14 hESC cultures, reaggregated *in vitro* and then transplanted under the kidney capsule of nude mice as in Figure 2. The grafts and spleens were harvested and analyzed (A, F) 12 and (C–E, G, H) 24 weeks (w) later. (A–E) T cell development in the grafts was analyzed. Representative flow cytometric profiles of (A, B) CD4 and CD8 DP, and SP, and (C) the number of T cells in the EpCAM^+^ and EpCAM^−^ cell grafts. Representative flow cytometric profiles of (D) the expression of αβ and γδ TCR by CD3^+^ T cells, and (E) CD4^+^Foxp3^+^ regulatory T cells in the EpCAM^+^ cell grafts. The number of (F, G) CD4 and CD8 T cells, as well as (H) CD4^+^Foxp3^+^ regulatory T cells in the spleens of the EpCAM^+^ and EpCAM^−^ cell-transplanted mice were analyzed by flow cytometry. Untreated nude mice were used as controls. The data are representative of 3 independent experiments with 5–6 mice per group. * p < 0.05 compared with untreated or EpCAM^−^ cell-treated nude mice.

Since the major function of TECs is to support T cell development, we assessed T cells in the hESC-EpCAM^+^ and EpCAM^−^ cell grafts over time. We found that CD4 and CD8 double positive (DP) and single positive (SP) T cells were generated in the hESC-EpCAM^+^ grafts, but not in the hESC-EpCAM^−^ grafts (Figure 3A). Importantly, T cell development in the EpCAM^+^ grafts was maintained through at least week 24 (Figure 3B, C). Analysis of the expression of αβ and γδ TCR by CD3^+^ T cells in the EpCAM1^+^ grafts showed that more than 90% of the cells were αβ TCR T cells (Figure 3D and Supplemental Fig. 3). CD4^+^Foxp3^+^ regulatory T cells were also observed in the EpCAM^+^ cell-transplanted mice (Figure 3E and Supplemental Fig. 3). These results suggest that the hESC-TEPs/TECs can attract mouse T cell precursors to the grafts and support their development into T cells.

In normal animals, T cells usually migrate to peripheral lymphoid organs after their maturation in the thymus. We then examined the percentage and number of CD4 and CD8 T cells in the spleen. There were only a few T cells in the untreated or hESC-EpCAM^−^ cell-transplanted nude mice (Figure 3F–H). In contrast, a considerable number of CD4 and CD8 T cells were present in the spleens of the hESC-EpCAM^+^ cell-transplanted nude mice 12 weeks after the transplantation (Figure 3F), suggesting T cells in the grafts can migrate to the periphery. A large number of CD4 and CD8 T cells, as well as CD4^+^Foxp3^+^ regulatory T cells still existed in the spleens 24 weeks after the transplantation (Figure 3G, H), consistent with the data that T cells continued development in the grafts. Collectively, the presence of CD4 and CD8 SP T cells in the spleens and CD4 and CD8 DP and SP T cells in the grafts of the hESC-EpCAM^+^ cell-transplanted nude mice suggest that the hESC-TECs support de novo generation of T cells.

### T cells in the hESC-TEP-transplanted mice are functional

**Figure 4:**
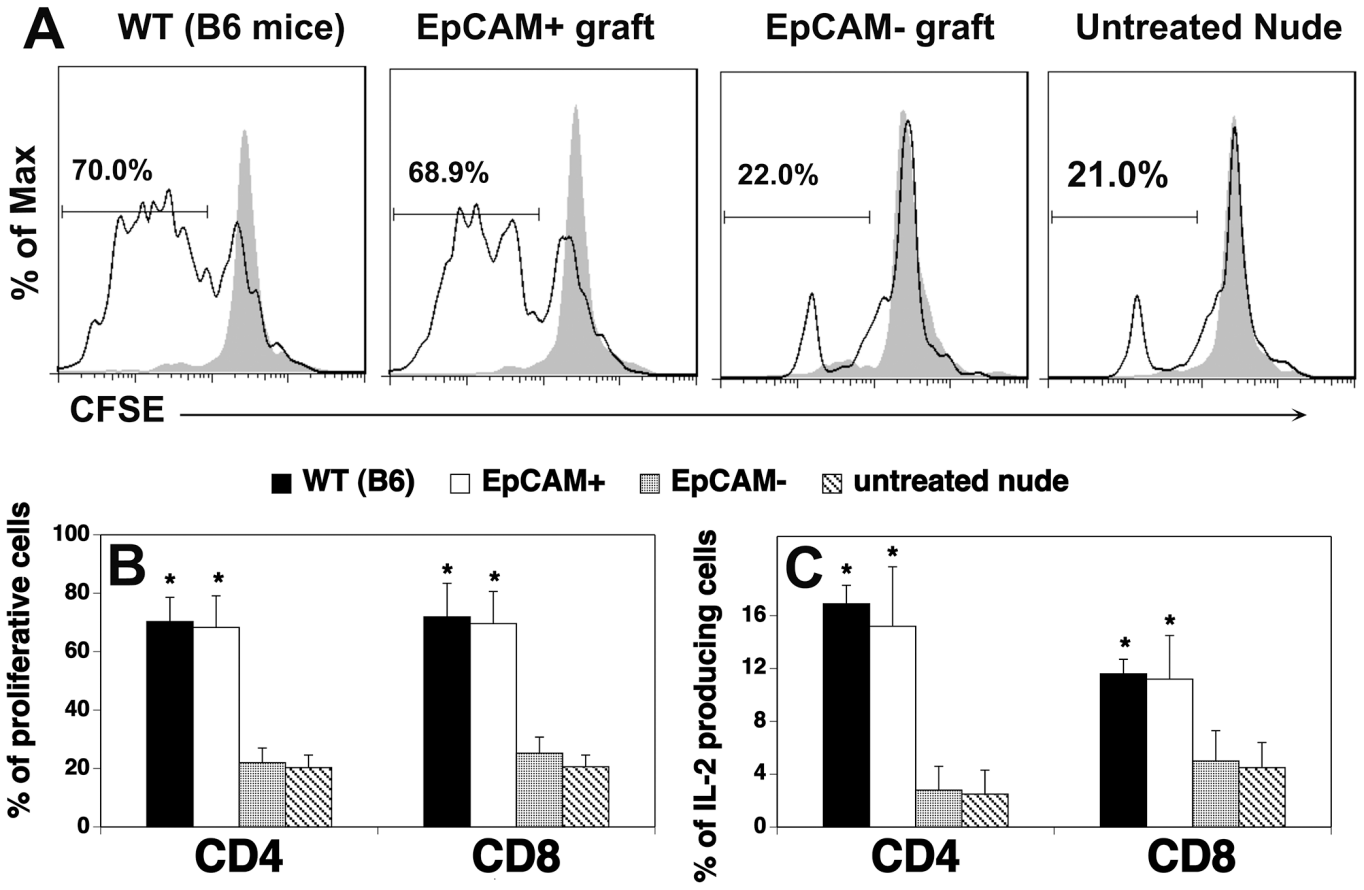
Peripheral T cells in the hESC-TEP-transplanted mice are functional. hESC-derived EpCAM^+^ and EpCAM^−^ cell reaggregates were transplanted under the kidney capsule of nude mice as in Figure 2. Untreated nude mice were used as controls. Twelve weeks later the spleens were harvested, and splenocytes were labeled with CFSE. Splenocytes were cultured in the presence of anti-CD3 and anti-CD28 antibodies (5 μg/ml) for 4 days. (A, B) The cells were stained with anti-CD4 and CD8 antibodies and analyzed for CFSE levels by CD4^+^ and CD8^+^ T cells. (A) Representative flow cytometric analysis of CFSE distribution of CD4^+^ T cells (nonstimulated cells are represented by gray filled lines), and (B) statistical analysis of T cell proliferation is shown. (C) The cells were stained with antibodies for cell surface markers and intracellular cytokine IL-2. The percentage of IL-2 positive cells in CD4^+^ and CD8^+^ T cells was determined by flow cytometry. The data are representative of 3 independent experiments with 5–6 mice per group. * p < 0.05 compared with untreated nude mice.

Functionally mature T cells are characterized by their ability to proliferate and produce cytokines in response to T cell receptor (TCR)-mediated signals^30^. To determine whether peripheral T cells in the hESC-TEP-transplanted mice were functional, we first examined T cell proliferation following TCR stimulation. To this end, splenocytes were loaded with carboxyfluorescein diacetate succinimidyl ester (CFSE), and then stimulated with anti-CD3 and CD28 antibodies. Cell proliferative ability was assessed by the loss of CFSE. As shown in Figure 4A and 4B, a significantly higher proportion of CD4^+^ and CD8^+^ T cells proliferated following anti-CD3 and anti-CD28 stimulation in the hESC-EpCAM^+^ cell-transplanted mice than in the hESC-EpCAM^−^ cell -transplanted or untreated nude mice. We then analyzed IL-2 producing T cells after TCR stimulation. Similarly, a significantly higher proportion of CD4^+^ and CD8^+^ T cells were able to produce IL-2 following TCR stimulation in the hESC-EpCAM^+^ cell-transplanted mice than in the control mice (Figure 4C). Taken together, these results suggest that the newly formed T cells in the hESC-TEP-transplanted mice are functional.

### Transplantation of hESC-TEPs and human hematopoietic precursors lead to the generation of functional human T cells

**Figure 5:**
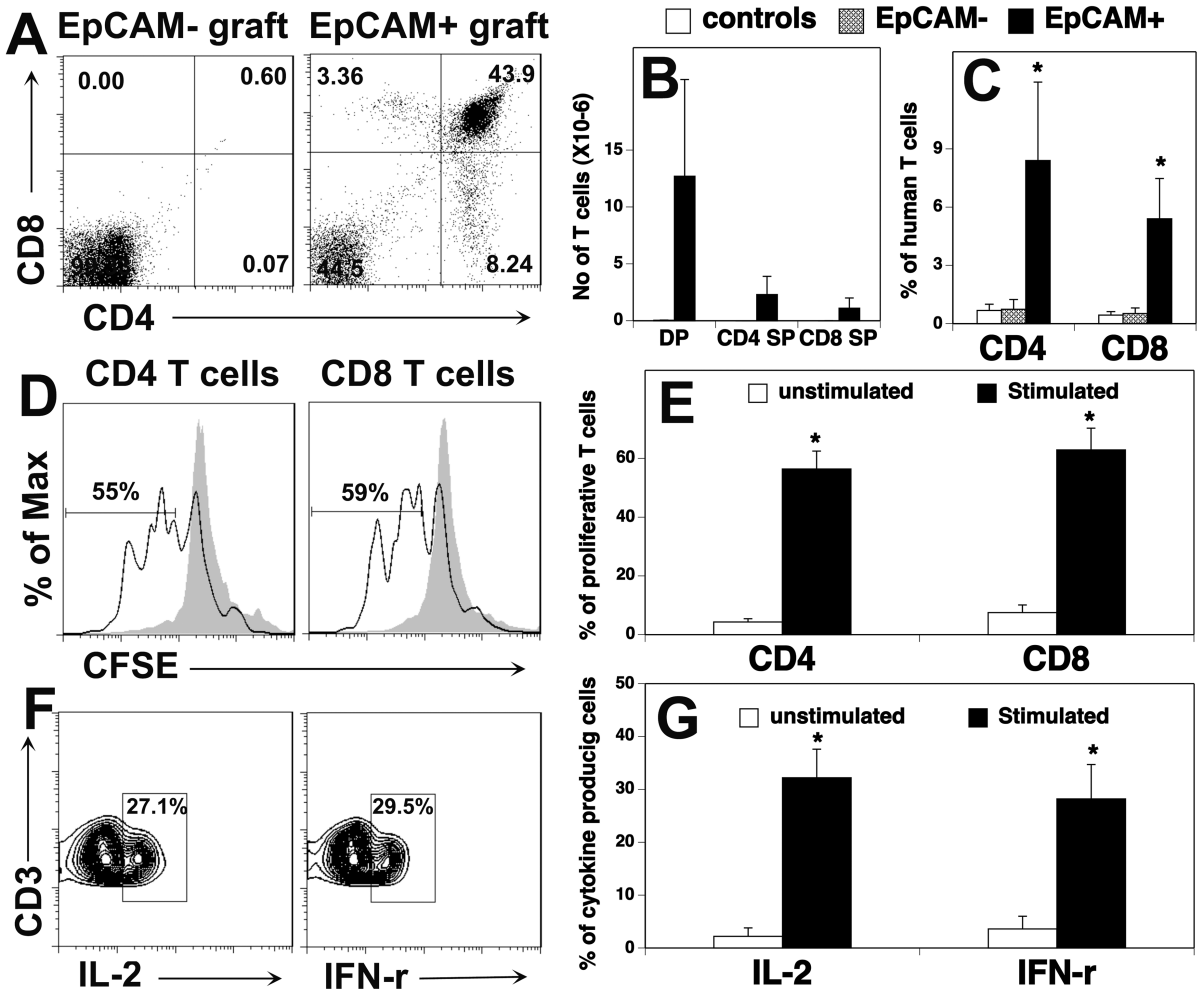
hESC-TECs support the development of functional human T cells. Four to six-week-old NSG mice were irradiated (2 Gy), transplanted under the kidney capsule with EpCAM^+^ or EpCAM^−^ cell reaggregates, and injected i.v. with human BM CD34^+^ cells. Mice that had been injected i.v. with human BM CD34^+^ cells only were used as a control. Twelve weeks later, the grafts, blood and spleens were harvested and analyzed. (A) Representative flow cytometric analysis of human CD4 and CD8 DP, and SP T cells, (B) the number of DP, CD4 SP and CD8 SP T cells in the grafts. (C) The percentage of human CD4 and CD8 T cells in the PBMCs. (D, E) Splenocytes from the EpCAM^+^ cell-transplanted mice were labeled with CFSE and cultured in the presence of Dynabeads® human T-Activator CD3/CD28 for 3 days. The cells were then stained with anti-huCD4 and huCD8 antibodies and analyzed for CFSE levels by CD4^+^ and CD8^+^ T cells. (D) Representative flow cytometric analysis of CFSE distribution of CD4^+^ or CD8^+^ T cells (unstimulated cells are represented by gray filled lines), (E) the percentage of proliferated T cells. (F, G) The splenocytes were cultured in the presence or absence of PMA and ionomycin. The cells were then stained with anti-huCD3 and human IL-2 or IFN-γ antibodies and analyzed by flow cytometry. (F) Representative flow cytometric profiles, and (G) the percentage of IL-2 or IFN-γ producing CD3^+^ T cells. The data are representative of 3 independent experiments with 5 mice per group. * p < 0.05 compared with control or unstimulated group.

Having shown that hESC-TECs supported the development of functional mouse T cells *in vivo*, we wanted to determine whether they were able to support human T cell development. To this end, irradiated NOD.Cg-Prkdcscid Il2rgtm1Wjl/SzJ (NSG) mice were transplanted with hESC-derived EpCAM^+^ cells or EpCAM^−^ control cells under the kidney capsule. These mice were also injected i.v. with human BM CD34^+^ hematopoietic precursors. Twelve weeks later, the grafts, peripheral blood (PB) and spleens were examined for the presence of human T cells. The hESC-EpCAM^+^ grafts contained human CD4 and CD8 DP and SP T cells, whereas hESC-EpCAM^−^ grafts did not (Figure 5A, B). Analysis of PB mononuclear cells (PBMCs) showed that very low percentages of human CD4 and CD8 SP T cells were present in the mice that had been transplanted with hESC-EpCAM^−^ cells and BM CD34^+^ hematopoietic precursors or BM CD34^+^ hematopoietic precursors only (Figure 5C), indicating that the thymus in the NSG mice supported low levels of human T cell development. In contrast, significantly higher percentages of human CD4 and CD8 SP T cells were present in the PBMCs of the hESC-EpCAM^+^ cell-transplanted mice (Figure 5C), consistent with the data that human T cell developed in the grafts. Similar results were obtained when the spleens were analyzed (data now shown).

To determine whether the human T cells are functional, splenic human T cells were examined for proliferation and cytokine-producing T cells after stimulation^31^. As shown in Figure 5D and E, a significantly higher proportion of human CD4 T cells or CD8 T cells in the hESC-EpCAM^+^ cell-transplanted mice were capable of proliferation after TCR stimulation, as compared to unstimulated cells. Similarly, a significantly higher proportion of human CD3 T cells in the hESC-EpCAM^+^ cell-transplanted mice were able to produce IL-2 and IFN-γ after stimulation (Figure 5F, G). These data suggest that the human T cells were functional. Collectively, hESC-TECs *in vivo* can also support the development of functional human T cells.

## Discussion

The thymus in mice initially arises from the endoderm of the third pharyngeal pouch^16^,^18^. We first induced the differentiation of hESCs into DE that was further induced to develop into TEPs. Although our protocol does not include separate steps that direct the differentiation of DE into the third PPE, or anterior foregut endoderm (AFE) and ventral pharyngeal endoderm (VPE)^13^,^14^, our kinetic analysis of the expression genes related to PPE and TEPs indicates that on days 9–11 the DE developed into the third PPE or VPE which then further developed into TEPs on day 14.

It has been reported that mesenchymal-epithelial interaction is important for early stages of thymic organogenesis^1^,^2^,^32^. The interaction is medicated, at least in part, by FGF7 and FGF10 produced by mesenchymal cells^1^,^2^,^33^. The BMP4 signaling pathway is also involved in the initial patterning of the thymus^16^. Therefore, we have included FGF7, FGF10, and BMP4 in our cultures for TEP differentiation. However, our data show that these factors are not sufficient to direct the differentiation of hESC-DE into TEPs, and that HOXA3 and FOXN1 are also required for the differentiation.

HOXA3, a member of the HOX family of transcription factors, has been proposed to be the earliest regulator for thymus organogenesis^17^,^34^. Mice homozygous for HOXA3 deletion are athymic^34^. FOXN1 is a member of the winged helix/forkhead box transcription factor family. A loss-of-function mutation in FOXN1 in mice, rats and humans results in the display the ‘nude’ phenotype, which is characterized by congenital athymia and hairlessness^35^. Many studies have shown that FOXN1 plays a critical role in TEC development^17^,^18^,^19^,^36^. It has been reported that the thymic rudiment in nude mice resembles respiratory epithelium, suggesting that FOXN1 may also be involved in the specification of a thymic developmental fate of the 3^rd^ PPE^37^. Because both the *FOXN1* and *HOXA3* genes in human and mouse are highly conserved in sequence and function^17^,^19^,^38^,^39^, we have used the murine forms of rFOXN1 and rHOXA3 in our studies. It was anticipated they would be functional in human cells. Indeed, our data showed that the addition of these two molecules significantly enhanced the generation of hESC-TEPs. The role of HOXA3 and FOXN1 in hESC-TEP generation remains to be further defined. It is possible that HOXA3 and FOXN1 induce the differentiation of hESC-DE into TEPs and/or promote the survival of hESC-TEPs.

Despite the addition of rFOXN1 protein to our culture system and induced endogenous expression of FOXN1, the hESC-TEPs could not spontaneously differentiate into TECs *in vitro*. However, when transplanted *in vivo*, the hESC-TEPs were able to further develop into TECs. It has been shown that, in addition to the intrinsic factor FOXN1, extrinsic signals from developing T cells are also important for TEC development^2^,^7^,^25^,^40^,^41^,^42^. It is possible that hESC-TEPs that express the chemokine CCL25 and other chemokines attract T cell precursors from the blood. TEPs interact with the T cell precursors to develop into TECs that, in turn, support the development of T cells from the T cell precursors.

In summary, we have developed a new protocol to efficiently generate TEPs from hESCs *in vitro*. Transplantation of purified hESC-TEPs results in the development of hESC-TECs that support long-term development of mouse T cells and a higher level of engraftment of human T cells. Our studies have important implications for the treatment of patients with primary and secondary T cell immunodeficiency.

## Methods

### Mice

NU/J nude and NSG mice were purchased from Jackson Laboratory. Mice were housed, treated, and handled according to protocols approved by the University of Connecticut Animal Care and Use Committee.

### Molecular cloning and expression of rHoxa3 protein

HOXA3-mCherry containing pSecTag2A vector was kindly provided by Dr. Kimberly A. Mace in the University of Manchester^22^. To obtain rHOXA3 without mCherry, the *Hoxa3* gene was amplified from the vector using primers 5′-GGTACCGAGCTCGGATCCAATGCAAAAAGCGACCTACTAC-3′ and

5′- GAGTTTTTGTTCGGGCCCCAGGTGGGTGAGCTTGGGCG-3′. The PCR product was cloned into the pSecTag2A vector (Invitrogen, Carlsbad, CA) using the In-Fusion HD Cloning Kit (Clontech Laboratories, Mountain View, CA) according to the manufacture's instruction. The pSecTag2A vector containing the *Hoxa3* gene was confirmed by DNA sequencing, and then transfected into CHO-S cells (Invitrogen). rHOXA3 or rHOXA3-mCherry was collected from the supernatant of the CHO-S cells that had been transfected with the pSecTag2A vector containing the *Hoxa3* or *Hoxa3-mCherry* gene, and verified by Western blot using a HOXA3 antibody. We used equimolar amounts of rHOXA3 and rHOXA3-mCherry in our initial studies, and obtained similar results (data not shown).

### Cell culture

Undifferentiated hESCs (H9 and CT2) were maintained on mouse embryo fibroblast feeder layers (GlobalStem, Gaithersburg, MD) in DMEM/F12 supplemented with 20% Knockout serum replacement (Invitrogen), 1 mM nonessential amino acids, Glutamax, penicillin/ streptomycin, 0.55 mM 2-mercaptoethanol (Invitrogen) and 4 ng/ml recombinant human FGF2 (R&D Systems, Minneapolis, MN). For the differentiation of hESCs into DE, the cells (4 × 10^3^ cells/well) were cultured in the medium from the Human Pluripotent cell-derived Endoderm Differentiation Kit (R&D Systems) according to the manufacturer's instructions for 4 days. For the differentiation of hESC-DE into TEPs, the cells were cultured in the presence of human FGF7 (20 ng/ml), FGF10 (20 ng/ml), EGF (50 ng/ml), BMP-4 (5 ng/ml) (R&D Systems or Pepro Tech, Rocky Hill, NJ), 0.5 μM all-trans RA (Sigma-Aldrich, St. Louis, MO), rFOXN1 (100 ng/ml) and rHOXA3 protein (200 ng/ml),

### Flow cytometry analysis

Cells were stained with the fluorochrome-conjugated antibodies as described^43^,^44^. For intracellular staining, the cells were first permeabilized with a BD Cytofix/Cytoperm solution for 20 minutes at 4°C. Direct or indirect staining of fluorochrome-conjugated antibodies included: mouse CD4, mouse CD8, mouse T cell receptor (TCR)β, TCR γδ, FoxP3, mouse or human (hu) interleukin-2 (IL-2), huCD4, CD8, huCD45, huCD3, huEpCAM, interferon (IFN)-γ, and HLA-DR (BioLegend or BD Biosciences, San Diego, CA), k5, Delta, and CCL25 (Santa Cruz Biotechnology, Santa Cruz, CA), k8 (US Biological, Swampscott, MA), AIRE, SOX17 (Millipore, Billerica, MA), fluorescein isothiocyanate (FITC), or phycoerythrin (PE) labeled anti-rat, or rabbit IgG (BD Biosciences). The samples were analyzed on a FACSCalibur (Becton and Dickinson Company). Data analysis was performed using FlowJo software (Ashland, OR).

### qRT-PCR

Total RNA from cells was isolated from cells, and cDNA was synthesized as described^45^. qRT- PCR was performed with the Power SYBR green mastermix (Applied Biosystems, UK) using the 7500 real-time PCR system (Applied Biosystems, UK). After normalization to *GAPDH*, samples were plotted relative to undifferentiated hESCs or day 4 hESC-derived DE. Human fetal thymus (HFT, obtained from Advanced Bioscience Resource) was used as a positive control. Primers are summarized in Supplemental Table 1.

### Western blot

Proteins were loaded on a 10% SDS-PAGE, transferred to a polyvinylidene fluoride membrane, and then incubated with following antibodies: anti-HOXA3 (Abcam, Cambridge, MA), FOXN1 (Thermo Scientific, or Fitzgerald, Acton, MA), PAX-1, or PAX-9 (Santa Cruz Biotechnology)^46^,^47^, and HRP conjugated secondary antibody. The membrane with proteins and antibodies was developed with Super Signal® West Pico chemiluminescent Substrate (Thermo Scientific).

### Immunomagnetic cell separation

Single-cell suspensions from ESC-derived cells were stained with PE labelled anti-huEpCAM antibody, washed, and stained with anti-PE MicroBeads (Miltenyi Biotec, Auburn, CA). EpCAM^+^ and EpCAM^−^ cells were selected using a magnetic-activated cell sorter immunomagnetic separation system (Miltenyi Biotec).

### Kidney capsule grafting and bone marrow transplantation

Purified hESC-derived EpCAM^+^ or EpCAM^−^ cells (1−2 × 10^6^) were subjected to reaggregate cultures for 24–48 hours, as described^12^. Four to six-week-old NU/J nude and NSG mice were used as recipients. The NU/J nude mice were injected i.p. with 25 μl of anti-asialo-GM1 (eBioscience, San Diego, CA) on day −1^29^, irradiated (3 Gy) and transplanted under the kidney capsule with the solidified reaggregate of the hESC-derived cells on day 0. NSG mice were irradiated (2 Gy), transplanted under the kidney capsule with the solidified reaggregate of the hESC-derived cells, and injected i.v. with human bone marrow CD34^+^ cells (1 × 10^5^ cells/mouse, obtained from AllCells, LLC, Alameda, CA). Twelve to 24 weeks after implantation, the grafts, spleens and blood were harvested and analyzed.

### Immunohistology and confocal microscopy

Immunohistological analysis of grafted thymus tissues was performed according to a modified protocol^12^. Briefly, tissues were incubated in 4% paraformaldeyde for 4 hours followed by incubation in 30% sucrose solution overnight. The tissues were embedded in OCT medium, snap frozen, and subsequently cut into 5 micrometer sections. The sections were stained with anti K8 monoclonal antibody (clone: CAM5.2, BD Biosciences) and rabbit anti K5 antibody (Abcam, Cambridge, MA), followed by FITC-conjugated anti-mouse IgG (Sigma) and AlexaFluor-546-conjugated goat anti-rabbit IgG (Invitrogen). The cells were observed under a Nikon A1R confocal microscope (Nikon, Kanagawa, Japan).

### T cell proliferation assays

Splenocytes were stained with 5 μM CFSE (Invitrogen) for 15 min. at 37°C. The cells were then cultured in a 96-well plate (4 × 10^5^ cells/well) in the presence of 5 µg/ml of plate-bound anti-mouse CD3 and anti-mouse CD28 antibodies or Dynabeads® human T-Activator CD3/CD28 (Invitrogen) for 3 days according the manufacture's instructions. The cells were then stained with anti-CD4 and CD8 antibodies and analyzed for CFSE levels by flow cytometry.

### Statistical analysis

P-values were based on the two-sided Student's T test. A confidence level above 95% (p < 0.05) was determined to be significant.

## Supplementary Material

Supplementary Information Supplemental Data
