# Supplementary material for: Efficient in vitro generation of functional thymic epithelial progenitors from human embryonic stem cells
Source: Sci Rep. 2015 Jun 5;5:9882. doi: 10.1038/srep09882 (PMC4456731; doi:10.1038/srep09882)
Supplement: Supplementary Information — Supplemental Data [file srep09882-s1.pdf]

Supplemental Information

Efficient *in vitro* generation of functional thymic epithelial progenitors from human embryonic stem cells

Min Su, Rong Hu, Jingjun Jin, Yuan Yan, Yinhong Song, Ryan Sullivan,

and Laijun Lai

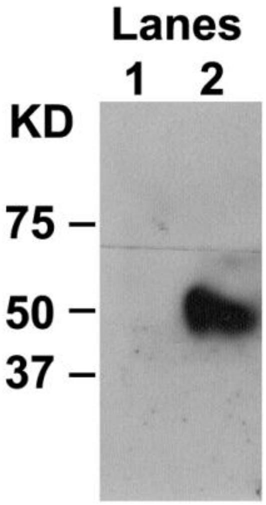

Supplemental Figure 1. Western blot analysis of rHOXA3 protein. Supernatant of the CHO-S cells that had been transfected with pSecTag2A vector without the Hoxa3 gene (lane 1) or with the Hoxa3 gene (lane 2) was examined by Western blot with a HOXA3 antibody.

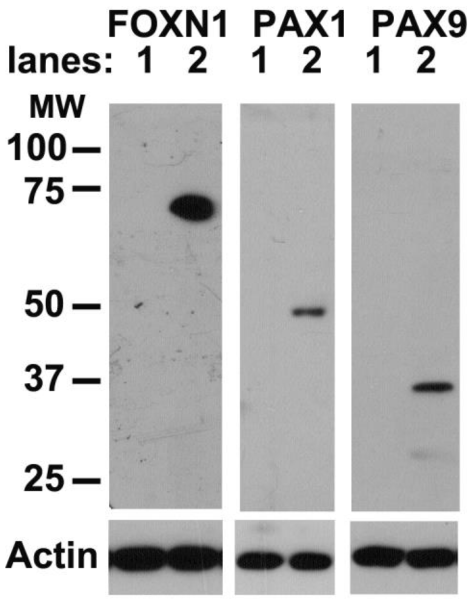

Supplemental Figure 2. Western blot analysis of FOXN1, PAX1, and PAX9 by day 14 hESC-derived cells. hESC-DE was cultured with BFFER, rFoxN1 and rHoxa3 as in Figure 1. hESC-DE (lane 1) and day 14 hESC-derived cells (lane 2) were examined for the expression of FOXN1, PAX1, and PAX9 proteins. Actin was used as a loading control.

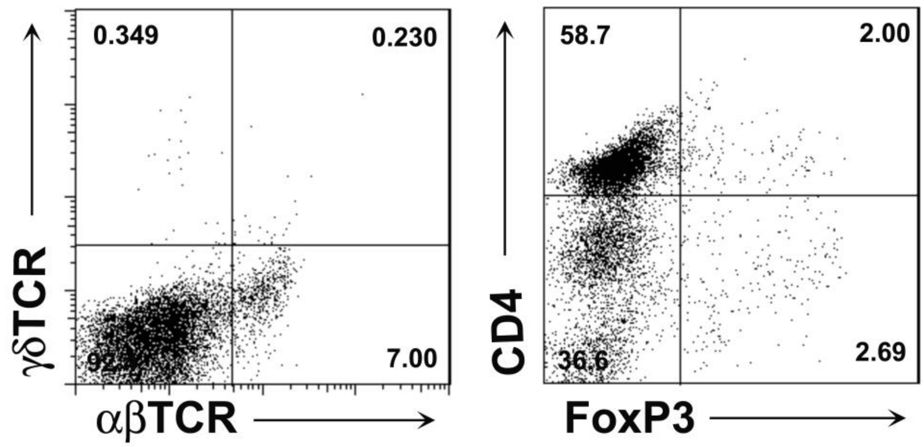

Supplemental Figure 3. EpCAM^{+} cells were purified from day 14 hESC cultures, reaggregated *in vitro* and then transplanted under the kidney capsule of nude mice as in Figure 3. The grafts were analyzed 24 weeks later. Representative flow cytometric profiles of the expression of αβ and γδ TCR, as well as CD4 and Foxp3 by ungated cells in the grafts.

Supplemental Table 1. Primer sequences used for qRT-PCR

Gene	Forward Primer	Reverse Primer
FOXA2	AAGATGGAAGGGCACGAG	TTCATGTTGCTCACGGAG
HOXA3	GCCAATCTGCTGAACCTC	ATGCCCTTGCCCTTCTGA
EYA1	AAGCCAGTTCAGATGTTG	ATTCACTACTACCACTCAGA
PAX1	GCAGTGAATGGGCTAGAGAAAC	ACGGCAGAGAGGGTGGAG
PAX9	TGTGTCAGCATCCAGCAT	TCCAGCAACATAACCAGAAG
FOXN1	TTTATGACGGAGCACTTT	CTTGTTGAGGGATAGGTT
GSC	ATCAGAGGAGTCGGAGAA	CCAAATCGCTTTTACCTTC
TBX1	ACGACAACGGCCACATTATTC	CCTCGGCATATTTCTCGCTATCT
GAPDH	TGCACCACCAACTGCTTAGC	GGCATGGACTGTGGTCATGAG
